# Preoperative ICG fluorescence marking improves lymph node retrieval and survival in laparoscopic gastrectomy for gastric cancer

**DOI:** 10.3389/fonc.2025.1606893

**Published:** 2025-07-21

**Authors:** Weijun Yang, Wubin Zheng, Jianning Dong, Xuesong Shi, Yongjun Nai

**Affiliations:** Department of general surgery, Nanjing First Hospital, Nanjing Medical University, Nanjing, China

**Keywords:** indocyanine green, fluorescence imaging, laparoscopic gastrectomy, gastric cancer, tumor localization, lymphadenectomy

## Abstract

**Background:**

While prior studies have suggested the potential benefits of preoperative indocyanine green (ICG) fluorescence marking in laparoscopic gastrectomy, few have evaluated its long-term oncologic impact in large real-world cohorts. This study aimed to validate the clinical utility of preoperative ICG marking by assessing its effects on lymph node retrieval and 3-year survival outcomes in gastric cancer patients.

**Methods:**

We retrospectively analyzed resectable gastric cancer patients who underwent laparoscopic gastrectomy at Nanjing First Hospital between January 2020 and December 2021. Patients were allocated to an ICG or non-ICG group. In the ICG group, 1.25 mg/mL ICG was endoscopically injected around the tumor 12 to 24 hours prior to surgery, with near-infrared imaging guiding tumor resection and lymph node (LN) dissection. Propensity score matching yielded 168 matched pairs. Primary outcomes were LN yield and tumor localization accuracy; secondary outcomes included operation time, blood loss, and 3-year disease-free survival (DFS) and overall survival (OS). Multivariable Cox regression analysis was used to identify independent prognostic factors.

**Results:**

The ICG group demonstrated a significantly higher mean LN yield (46.4 ± 8.5 vs. 42.6 ± 11.5, *P*<0.01), lower noncompliance (31.0% vs. 49.4%, *P*<0.01), shorter operation time (200.0 ± 11.4 vs. 210.4 ± 11.6 minutes, *P*<0.01), and lower intraoperative blood loss (26.9 ± 8.7 vs. 31.3 ± 9.2 mL, *P*<0.01). The 3-year DFS and OS rates were significantly improved in the ICG group (DFS: 74% vs. 60%; OS: 80% vs. 66%; log-rank *P*<0.01). Multivariable Cox regression confirmed that ICG use was independently associated with improved DFS (HR=0.44, 95% CI: 0.28–0.71, *P*<0.01) and OS (HR=0.44, 95% CI: 0.25–0.76, *P*<0.01).

**Conclusions:**

Preoperative ICG fluorescence marking is a safe and effective adjunct in laparoscopic gastrectomy for gastric cancer, enhancing surgical efficiency and long-term outcomes.

## 1 Introduction

Gastric cancer is the fifth most common malignancy and the fourth leading cause of cancer-related deaths worldwide, with approximately 1 million new cases and over 768,000 deaths in 2020 (1). Notably, its incidence varies significantly across regions, with East Asia—especially China, Japan, and South Korea—bearing the highest disease burden (2). In China alone, gastric cancer accounts for roughly 40% of global cases, underscoring the urgent need for effective treatment strategies (3).

Surgical resection remains the primary curative treatment for gastric cancer, and laparoscopic gastrectomy has gained widespread acceptance due to its minimally invasive nature, lower postoperative complication rates, and faster recovery times (4). However, ensuring precise tumor localization and performing adequate lymphadenectomy—particularly achieving the diagnostic threshold of ≥30 retrieved lymph nodes—continue to pose significant challenges in laparoscopic procedures, directly impacting staging accuracy and oncologic outcomes. (5, 6).

Indocyanine green (ICG) fluorescence imaging has emerged as a valuable tool in gastrointestinal surgery, providing real-time visualization of tumors and lymphatic pathways (7). Preoperative endoscopic injection of ICG enables precise intraoperative identification of tumor margins and enhanced lymph node mapping, potentially improving surgical accuracy and reducing complications (8). Despite these promising advantages, the clinical utility of ICG fluorescence imaging for preoperative tumor marking in laparoscopic gastrectomy remains under investigation. While prior studies have suggested potential improvements in surgical quality, data on survival benefits—particularly in real-world Chinese populations—are limited.

Therefore, this study aims to confirm the efficacy and safety of preoperative ICG fluorescence marking in laparoscopic gastrectomy for gastric cancer, focusing on its impact on surgical precision, lymph node retrieval, and long-term survival outcomes. By employing propensity score matching (PSM) and reporting longer-term oncologic outcomes in a large Chinese patient cohort, this study seeks to validate and expand upon prior findings, providing robust evidence for the clinical utility of ICG in real-world surgical settings.

## 2 Materials and methods

### 2.1 Patients

This retrospective study included adult patients (≥18 years) with histologically confirmed, resectable gastric adenocarcinoma (T1-T4a) who underwent laparoscopic gastrectomy at Nanjing First Hospital between January 2020 and December 2021. Patients were eligible if preoperative staging indicated resectable disease, they were scheduled for laparoscopic radical gastrectomy with D2 lymphadenectomy, and had received no prior neoadjuvant chemotherapy or radiotherapy. Exclusion criteria were: (1) distant metastases, (2) severe comorbidities precluding surgery, and (3) allergy to ICG.

Preoperative assessment included enhanced computed tomography (CT) for tumor staging and localization. All patients also underwent diagnostic upper gastrointestinal endoscopy with biopsy to confirm histopathological diagnosis and guide surgical planning.

### 2.2 ICG fluorescence marking protocol

In the ICG group, a solution of 1.25 mg/mL ICG (Dandong Yichuang Pharmaceutical Co) was prepared using sterile water, and 0.5 mL was endoscopically injected into the submucosal layer at four quadrants around the tumor, totaling 2.5 mg, 12 to 24 hours before surgery. The submucosal injection was confirmed in real time by observing mucosal elevation during the slow injection. All injections were performed under direct endoscopic visualization using a standard 23G endoscopic needle, which allowed precise control of the injection depth and location around the tumor margins. Near-infrared (NIR) fluorescence imaging was utilized intraoperatively to guide tumor resection and LN dissection. The imaging system (NOVADAQ, Stryker, US) enabled seamless switching between visible light and NIR fluorescence to optimize tumor localization and lymphadenectomy. Fluorescence imaging was also used post-dissection to verify the completeness of LN dissection.

### 2.3 Surgical procedure

All surgeries were performed by a dedicated gastrointestinal oncology team composed of three senior surgeons, each with over 10 years of experience in laparoscopic gastrectomy. All patients underwent laparoscopic gastrectomy with D2 lymphadenectomy following standard oncological principles. The extent of resection (total or subtotal gastrectomy) was determined based on tumor location and size. In the ICG group, the fluorescence signal was used to guide both tumor localization and LN dissection, whereas the non-ICG group relied on conventional intraoperative techniques, including palpation and preoperative imaging. In the non-ICG group, intraoperative tumor localization and lymph node dissection were guided primarily by systematic palpation of the gastric wall and regional lymph node basins. Experienced surgeons palpated the stomach to identify the tumor margins and assessed the consistency and enlargement of lymph nodes manually. This tactile feedback was used to determine the extent of resection and the lymph node stations requiring dissection. Palpation was performed according to a standardized protocol to ensure thorough examination of all relevant anatomical regions.

Postoperative adjuvant chemotherapy was administered according to national clinical guidelines based on the final pathological stage and patient condition (9).

### 2.4 Outcome measures

The primary outcomes were the number of LNs retrieved and tumor localization accuracy. The current American Joint Committee on Cancer (AJCC) Staging manual recommends that the removal of ≥30 regional LNs is desirable (10). LN dissection noncompliance was defined as the absence of LNs from more than 1 LN station that should have been excised.

Secondary outcomes included disease-free survival (DFS) and overall survival (OS) at three years. In addition, total operation time and intraoperative blood loss were analyzed. Postoperative follow-up was conducted every 3 months. At each visit, patients underwent clinical examination, routine blood tests including tumor markers, and contrast-enhanced abdominal CT. Upper gastrointestinal endoscopy was performed annually. Any suspected recurrence was confirmed through imaging, histopathological biopsy, or multidisciplinary team (MDT) evaluation. Survival status and recurrence events were collected from the hospital’s database. Patients who were alive at the time of the last follow-up or lost to follow-up before death were considered censored in the survival analysis.

### 2.5 Statistical analysis

To minimize selection bias, a PSM analysis was performed to balance baseline characteristics between the ICG and non-ICG groups. Propensity scores were calculated using a multivariable logistic regression model based on key covariates, including age, sex, body mass index (BMI), ECOG performance statues, tumor location, lymph vascular invasion, tumor size, tumor stages, and AJCC stage. Nearest-neighbor matching without replacement was conducted using a caliper width of 0.2 standard deviations of the logit of the propensity score.

Continuous variables were compared using the Student’s t-test or Mann-Whitney U test, while categorical variables were analyzed using the chi-square test or Fisher’s exact test. Kaplan-Meier survival analysis with the log-rank test was used to compare DFS and OS between the ICG and control groups. Multivariate Cox proportional hazards regression analysis was performed to identify independent prognostic factors for DFS and OS, adjusting for potential confounders, including age, sex, tumor stage, and lymph node involvement. A *P* value *<* 0.05 was considered statistically significant. Data were analyzed using SPSS version 22.0 (IBM Corp., Armonk, NY, USA).

## 3 Results

### 3.1 Baseline characteristics

**Figure 1:**
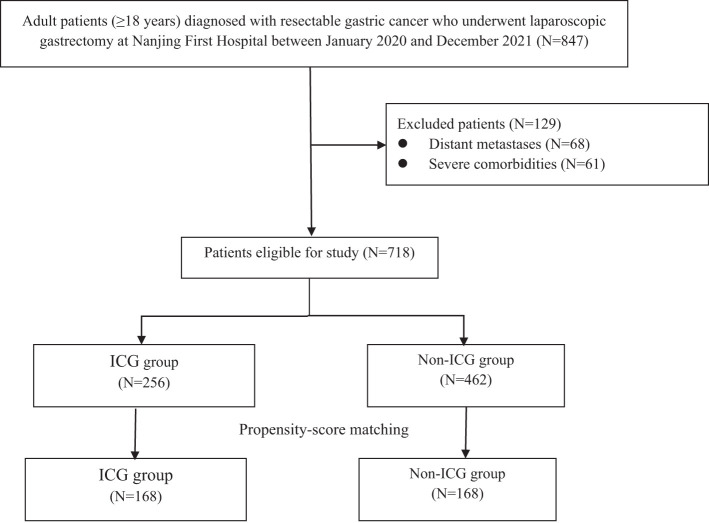
Patients’ selection flow.

A total of 847 patients diagnosed with gastric cancer were initially enrolled. After excluding 129 cases, 718 patients remained, with 256 (35.7%) receiving ICG. Propensity score matching yielded 168 matched pairs (336 patients) for subsequent analysis (**Figure 1**). The baseline characteristics of the matched cohorts are summarized in **Table 1**, and no significant differences were observed in age, sex, BMI, ECOG performance status, tumor location, lymphovascular invasion, tumor size, tumor stage, or AJCC stage (all P>0.05).

**Table 1:** Baseline characteristics of the ICG group and non-ICG group.

Variables	ICG group (n=168)	Non-ICG group (n=168)	*P* value
Age, years	58.1 ± 5.7	58.5 ± 5.8	0.48
BMI, kg/m^2^	22.9 ± 1.7	23.1 ± 2.2	0.26
Gender			0.65
Male	106 (63.1%)	102 (60.7%)	
Female	62 (36.9%)	66 (39.3%)	
ECOG performance statues			0.57
0	140 (83.3%)	136 (81.0%)	
1	28 (16.7%)	32 (19.0%)	
Tumor Location			0.67
Upper	39 (23.2%)	34 (20.2%)	
Middle	34 (20.2%)	31 (18.5%)	
Lower	95 (56.5%)	103 (61.3%)	
Lymphvascular invasion			0.37
Negative	106 (63.1%)	98 (58.3%)	
Positive	62 (36.9%)	70 (41.7%)	
Size, cm			0.44
≤3	78 (46.4%)	71 (42.3%)	
>3	90 (53.6%)	97 (57.7%)	
cT stage			0.99
cT1	46 (27.4%)	45 (26.8%)	
cT2-cT3	92 (54.8%)	92 (54.8%)	
cT4	30 (17.9%)	31 (18.5%)	
cN stage			0.44
cN0	73 (43.5%)	80 (47.6%)	
cN+	95 (56.5%)	88 (52.4%)	
pT stage			0.74
pT1	62 (36.9%)	65 (38.7%)	
pT2-T4a	106 (63.1%)	103 (61.3%)	
pN stage			0.40
pN0	79 (47.0%)	72 (42.9%)	
pN1	18 (10.7%)	27 (16.1%)	
pN2	30 (17.9%)	24 (14.3%)	
pN3	41 (24.4%)	45 (26.8%)	
AJCC stage			0.35
I	75 (44.6%)	84 (50.0%)	
II	45 (26.8%)	34 (20.2%)	
III	48 (28.6%)	50 (29.8%)	

### 3.2 Surgical outcomes

As shown in **Table 2**, the ICG group had a significantly higher mean LN yield (46.4 ± 8.5) compared to the non-ICG group (42.6 ± 11.5, *P*<0.01). Notably, all patients (100%) in the ICG group achieved the recommended LN yield (≥30 nodes), whereas only 87.5% (n=147) of the non-ICG group met this threshold (*P*<0.01). When stratified by anatomical region, the number of LNs retrieved was significantly higher in both the D1 (25.5 ± 4.7 vs. 23.5 ± 6.3, *P*<0.01) and D2 (20.8 ± 3.8 vs. 19.1 ± 5.1, *P*<0.01) stations in the ICG group. In terms of lymph node dissection accuracy, the ICG group showed a significantly lower noncompliance rate than the non-ICG group (31.0% vs. 49.4%, *P*<0.01). These results suggest that preoperative ICG marking contributes not only to a higher total lymph node yield, but also to a more standardized and comprehensive lymphadenectomy.

**Table 2:** Primary and secondary outcomes.

Variables	ICG group (n=168)	Non-ICG group (n=168)	*P* value
Primary outcomes			
Total LN retrieved	46.4 ± 8.5	42.6 ± 11.5	<0.01
≥30	168 (100.0%)	147 (87.5%)	<0.01
<30	0	21 (12.5%)	
D1 station LNs retrieved	25.5 ± 4.7	23.5 ± 6.3	<0.01
D2 station LNs retrieved	20.8 ± 3.8	19.1 ± 5.1	<0.01
LNs dissection compliance			<0.01
Compliance	116 (69.0%)	83 (49.4%)	
Noncompliance	52 (31.0%)	85 (50.6%)	
Secondary outcomes			
Operation times, minutes	200.0 ± 11.4	210.4 ± 11.6	<0.01
Blood loss, ml	26.9 ± 8.7	31.3 ± 9.2	<0.01
With postoperative complication	37 (22.0%)	32 (19.0%)	0.50
Recurrence rate	23 (13.7%)	42 (25.0%)	<0.01
Mortality	32 (19.0%)	55 (32.7%)	<0.01

Operative efficiency was also improved: the total operation time was significantly shorter in the ICG group (200.0 ± 11.4 minutes vs. 210.4 ± 11.6 minutes, *P*<0.01) and intraoperative blood loss was reduced (26.9 ± 8.7 mL vs. 31.3 ± 9.2 mL, *P*<0.01). Postoperative complication rates were similar between the groups (22.0% vs. 19.0%, *P*=0.50).

### 3.3 Survival outcomes

**Figure 2:**
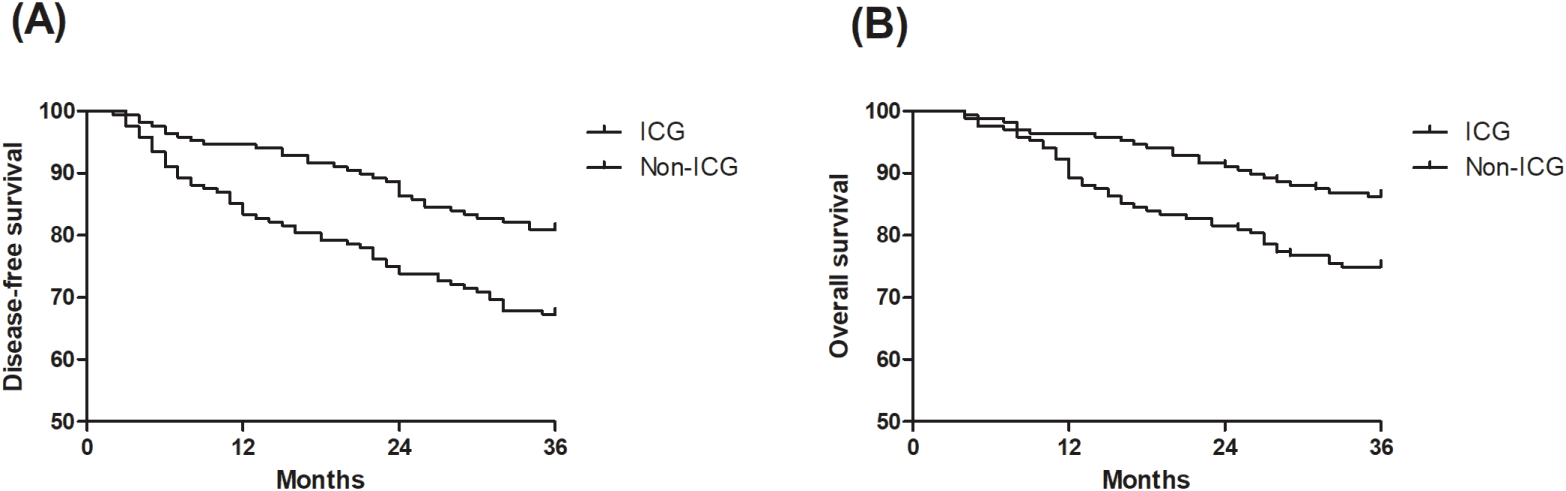
Survival analysis of the indocyanine green (ICG) group and non-ICG group. **(A)** comparing disease-free survival (DFS) between the ICG group and non-ICG group. **(B)** comparing overall survival (OS) between the ICG group and non-ICG group.

ICG fluorescence marking was associated with significantly better long-term outcomes. The 3-year recurrence rate was lower in the ICG group (13.7% vs. 25.0%, *P*<0.01), and mortality was reduced (19.0% vs. 32.7%, *P*<0.01). Kaplan-Meier curves (**Figure 2**) demonstrated significantly better three-year DFS (HR=0.53, 95% CI: 0.34-0.80; log-rank *P*<0.01) and OS (HR=0.51, 95% CI: 0.31-0.84; log-rank *P*<0.01) in the ICG group. Multivariable Cox regression analysis (**Table 3**) confirmed that ICG use was independently associated with improved DFS (HR=0.44, 95% CI: 0.28–0.71, *P*<0.01) and OS (HR=0.44, 95% CI: 0.25–0.76, *P*<0.01) after controlling for potential confounding factors (**Table 3**). Additionally, advanced AJCC stage (II and III) was a significant negative prognostic factor for both DFS and OS (both *P*<0.01).

**Table 3:** Multivariable Cox regression analyses for exploring the association of indocyanine green fluorescence (ICG) with disease-free survival (DFS) and overall survival (OS).

Variables	DFS			OS		
	HR	95% CI	*P*	HR	95% CI	*P*
ICG vs. non-ICG	0.44	0.28-0.71	<0.01	0.44	0.25-0.76	<0.01
Age	0.99	0.95-1.03	0.57	0.98	0.94-1.02	0.33
BMI	0.92	0.83-1.03	0.14	0.95	0.83-1.08	0.39
Gender						
Male	Ref.			Ref.		
Female	1.17	0.74-1.83	0.51	1.23	0.73-2.07	0.45
ECOG performance statues						
0	Ref.			Ref.		
1	1.11	0.63-1.95	0.72	1.03	0.54-1.96	0.93
Tumor Location						
Upper	Ref.			Ref.		
Middle	1.09	0.54-2.21	0.80	0.58	0.25-1.33	0.20
Lower	1.14	0.69-1.88	0.62	0.69	0.40-1.19	0.18
Lymphvascular invasion						
Negative	Ref.			Ref.		
Positive	0.86	0.58-1.27	0.46	1.05	0.68-1.64	0.82
Size, cm						
≤3	Ref.			Ref.		
>3	1.44	0.90-2.30	0.13	1.55	0.90-2.67	0.11
cT stage						
cT1	Ref.			Ref.		
cT2-cT3	1.01	0.59-1.73	0.96	1.14	0.62-2.09	0.67
cT4	0.81	0.40-1.63	0.55	0.98	0.45-2.15	0.96
cN stage						
cN0	Ref.			Ref.		
cN+	1.05	0.67-1.65	0.82	1.04	0.62-1.74	0.90
pT stage						
pT1	Ref.			Ref.		
pT2-T4a	0.79	0.50-1.26	0.33	1.03	0.60-1.75	0.92
pN stage						
pN0	Ref.			Ref.		
pN1	0.87	0.44-1.74	0.70	0.86	0.39-1.93	0.72
pN2	0.52	0.24-1.11	0.09	0.53	0.21-1.30	0.16
pN3	1.48	0.89-2.46	0.14	1.54	0.85-2.78	0.15
AJCC stage						
I	Ref.			Ref.		
II	6.65	2.98-14.84	<0.01	4.80	2.08-11.08	<0.01
III	14.84	7.18-30.65	<0.01	8.28	3.91-17.55	<0.01

## 4 Discussion

This study demonstrates that preoperative ICG fluorescence marking offers significant clinical benefits in laparoscopic gastrectomy for gastric cancer. In our cohort, the use of ICG improved intraoperative tumor localization, enhanced the extent of LN dissection, and was associated with favorable long-term outcomes. These findings add to the growing evidence supporting the integration of fluorescence imaging technologies in minimally invasive oncologic surgery.

### 4.1 Improved tumor localization and operative efficiency

Accurate intraoperative tumor localization remains a challenge in totally laparoscopic procedures, particularly for early-stage or non-palpable lesions. Our results show that preoperative submucosal ICG injection significantly enhanced visualization of tumor margins, facilitating precise proximal margin determination and surgical planning. This aligns with the retrospective study by Yoon and Lee (11), which demonstrated that ICG marking not only secured an oncologically safe proximal resection margin during totally laparoscopic distal gastrectomy but also reduced operative time by approximately 34 minutes. However, several challenges persist regarding tumor localization accuracy. These include variability in the depth and volume of ICG injection, timing of administration relative to surgery, and heterogeneity in fluorescence signal intensity due to factors such as tissue thickness and tumor characteristics (12, 13). Moreover, subjective interpretation of fluorescence images may contribute to inconsistency in surgical decision-making. Previous studies have highlighted these issues as significant barriers to widespread clinical adoption (14). To address these challenges, future research should focus on developing standardized protocols for ICG administration, including precise injection techniques, optimal dosing, and timing schedules. In addition, the integration of objective quantification methods for fluorescence intensity and real-time imaging guidance could improve localization accuracy and reproducibility. Multicenter studies employing such standardized approaches would help validate the clinical utility of ICG fluorescence marking and facilitate its broader implementation in clinical practice.

### 4.2 Enhanced lymphadenectomy and long-term oncologic outcomes

Adequate LN dissection is critical for accurate staging and improved disease control in gastric cancer. In our analysis, the ICG group yielded a significantly higher number of retrieved LNs compared to the non-ICG group. Notably, this increase in LN yield was observed uniformly across both D1 and D2 nodal stations, indicating that ICG fluorescence guidance enhances retrieval from anatomically diverse and potentially challenging regions. This observation aligns with the findings of a phase III randomized clinical trial by Chen et al. (15), where the mean LN count was substantially higher in the ICG group (50.5 vs. 42.0, *P*<0.001). Moreover, their study reported superior three-year overall survival (OS) and disease-free survival (DFS) in the ICG group, along with a lower recurrence rate (17.8% vs. 31.0%). These results strongly support the prognostic value of ICG-guided lymphadenectomy, suggesting that a more extensive and precise LN dissection can contribute to improved long-term outcomes. It is noted that the more comprehensive LN retrieval in the ICG group may contribute to potential “upstaging” by detecting additional metastatic nodes. Such upstaging could partly explain the improved survival outcomes observed, as more accurate staging facilitates tailored postoperative treatment and surveillance. Importantly, the addition of ICG did not alter the predefined anatomical clearance range. All patients in both groups underwent D2 lymphadenectomy according to standardized oncologic guidelines. ICG fluorescence imaging served as an intraoperative navigational aid, improving visualization and identification of lymphatic tissue, but did not expand or reduce the intended dissection field. Therefore, the observed increase in lymph node yield in the ICG group likely reflects enhanced precision and completeness of dissection rather than a modification of surgical extent.

In addition, a prospective cohort study by Wei et al. (16) further validated the oncologic safety and feasibility of ICG fluorescence imaging in laparoscopic radical gastrectomy. Patients in the ICG-assisted group not only exhibited a higher LN yield but also benefited from shorter operation and dissection times and reduced intraoperative blood loss. Although the 2-year OS and DFS did not differ significantly between groups in Wei et al.’s study, the observed procedural improvements mirror our findings and suggest that ICG guidance enhances surgical quality without compromising oncologic safety.

### 4.3 Mechanisms and implications for future practice

The potential mechanism behind the improved outcomes with ICG may be twofold. First, real-time fluorescence imaging allows for more accurate identification of tumor margins and lymphatic drainage pathways, facilitating a more comprehensive resection. Second, a more thorough LN dissection may reduce the residual micrometastatic burden, thus contributing to a lower recurrence rate and improved survival. Our findings that LN yield increased uniformly across nodal stations and the potential for upstaging underscore that ICG fluorescence imaging not only enhances surgical precision but also may improve pathological staging accuracy, which in turn guides optimal postoperative management. Although these mechanisms remain to be fully elucidated, our results, in conjunction with previous studies, support the notion that ICG fluorescence imaging can translate into meaningful oncologic benefits beyond technical facilitation.

### 4.4 Limitations and future directions

Despite the promising results, this study has several limitations. As a single-center retrospective analysis, our findings may be influenced by selection bias and center-specific surgical expertise. Although propensity score matching was employed to mitigate confounding, unmeasured variables might still affect the outcomes. Additionally, as detailed earlier, variability in injection depth, volume, timing, and subjective interpretation of fluorescence signals remains a major challenge. Future work should prioritize developing standardized ICG administration protocols and objective imaging quantification, supported by multicenter validation studies to facilitate clinical adoption. Finally, while our follow-up duration was sufficient to detect early recurrences, longer-term survival outcomes require further evaluation.

## 5 Conclusion

This study demonstrates that preoperative ICG fluorescence marking significantly improves operative efficiency, evidenced by shorter operation time and reduced intraoperative blood loss, without increasing postoperative complications, thus confirming its safety. Multivariable Cox regression further identified ICG use as an independent predictor of improved disease-free and overall survival, highlighting its oncological benefit. Notably, 100% of patients in the ICG group achieved the recommended lymph node yield (≥30 nodes), compared to 87.5% in the non-ICG group, underscoring its role in enhancing lymphadenectomy quality. These findings align with previous randomized and retrospective studies, reinforcing the value of ICG in improving both intraoperative decision-making and oncological outcomes. As ICG imaging becomes more widely accessible, future prospective multicenter trials are warranted to standardize its use and clarify its long-term impact on survival.

## Data Availability

The raw data supporting the conclusions of this article will be made available by the authors, without undue reservation.
